# Reduction of Incarcerated Hernia

**Published:** 1849-02

**Authors:** 


*Reduction of Incarcerated Hernia.* By M. Amussat.—The method adopted by M. A. which has often succeeded in causing reduction of incarcerated hernia, when other measures had failed, is the following: A board being first placed under the pelvis, in order to give a solid fulcrum to the efforts of the surgeon, both hands are applied to the tumour, exercising a moderate degree of pressure upon it; this pressure is gradually increased, by the super-position of the hands of the assistants over those of the operator. Thus the efforts can be uninterruptedly continued for a considerable length of time without fatigue to the surgeon, and often with the most satisfactory results.—*London Med. Times from Revue Med.*

